# Galphimine-B Standardized Extract versus Alprazolam in Patients with Generalized Anxiety Disorder: A Ten-Week, Double-Blind, Randomized Clinical Trial

**DOI:** 10.1155/2019/1037036

**Published:** 2019-01-30

**Authors:** Ofelia Romero-Cerecero, Ana Laura Islas-Garduño, Alejandro Zamilpa, Armando Herrera-Arellano, Enrique Jiménez-Ferrer, Jaime Tortoriello

**Affiliations:** ^1^Centro de Investigación Biomédica del Sur, Instituto Mexicano del Seguro Social (CIBIS-IMSS), Xochitepec, Morelos, Mexico; ^2^Facultad de Medicina de la Universidad Autónoma del Estado de Morelos, Cuernavaca, Morelos, Mexico

## Abstract

Galphimine-B (G-B), a compound isolated from* Galphimia glauca*, has been shown to possess important anxiolytic activity. In this study, we evaluated the effectiveness and tolerability of a G-B standardized extract (experimental treatment) that was administered daily for 10 weeks in patients with moderate or severe Generalized Anxiety Disorder (GAD). Alprazolam was used as control treatment and administered under the same conditions. A total of 167 patients were included. At the start of the study, the severe anxiety condition prevailed, with an average on the Hamilton Anxiety Scale of 35.1 ± 8.8 and 35.8 ± 8.1 points in the control and experimental groups, respectively. After the 10 weeks of administration, the average was reduced in the control group to 4.6 ± 6.5 points and in the experimental group to 3.5 ± 5.5 points. Therapeutic success in the control group was 85.7% and in the experimental group, 92.0%. A high proportion of patients (22.2%) treated with Alprazolam manifested daytime sleepiness, while in the group treated with the G-B standardized extract, daytime sleepiness was found in 4.7%. In conclusion, a G-B standardized extract demonstrated therapeutic effectiveness in patients with GAD, without exhibiting significant difference with Alprazolam, but showing fewer cases of daytime sleepiness. The trial was registered at http://clinicaltrials.gov by identifier: NCT03702803.

## 1. Introduction

Mental disorders have become a public-health problem because of their frequency and impact on society and the economy. The World Health Organization (WHO) has estimated that 450 million persons worldwide suffer from a mental disorder, one in four individuals will be affected by a mental disorder at some time during their lives, and it is estimated that one of every four families in the world has a member with some mental disorder [1]. In Mexico, the National Survey of Psychiatric Epidemiology reported that anxiety disorders were the most frequent (14.3%) of these among mental disorders [2].

Mental and neuropsychiatric diseases have been considered as disabling and could be able to produce more disability than general medical health problems [3], while it has been estimated that four of five patients with mental disorder do not receive the medical services they require [4]. It has also been pointed out that the prognosis for patients suffering from an anxiety disorder is less favorable than for patients with depression [5].

Triazolobenzodiazepines are modern molecules with potent hypnotic effects and short half-lives [6]. However, benzodiazepines, even considering the most recent of these, have disadvantages due to their adverse effects [7]. Azapirones and selective Serotonin Reuptake Inhibitors (SSRI), which present fewer adverse effects, possess important limitations because they show effectiveness in a low percentage of patients. In the initial phase of their administration, they can worsen anxiety and their anxiolytic effect is observed after 3 weeks of administration [8].

In Mexican Traditional Medicine, the plant species* Galphimia glauca* (popularly known as Corpionchi or Golden Bouquet) has been employed for many years as a “sedative or nervous tranquilizer” [9]. The traditional knowledge was utilized as a basis to initiate the scientific investigation of this species. In in vitro and in vivo models, it was demonstrated that the crude extract obtained from the aerial parts of* G. glauca* possesses effects that depress the excitability of the nervous system. Through bio-guided chromatographic separation, it was possible to isolate and structurally elucidate a new compound, a nor-seco-triterpene, which was given the name of Galphimine-B (G-B). By means of several pharmacological experiments, it has been possible to discover that the effect produced by G-B on the Central Nervous System (CNS), unlike benzodiazepines, is selective of dopaminergic neurons of the Ventral Tegmental Area (VTA) in rat brain and does not interact with the GABAergic system [10]. In addition, progress has been made in elucidating the mechanism of action of G-B: extracellular unitary records of the frequency of discharge of dopaminergic neurons have revealed that this compound has the ability to block the effect produced by glutamate on NMDA receptors. Moreover, interaction with the serotonergic system in the dorsal hippocampus has also been identified in rat brain [11].

Regarding clinical trials, phytopharmaceuticals, elaborated with the* G. glauca *extract and administered orally for 4 weeks in patients with GAD, were able to reduce anxiety in a significant manner. The extract's effectiveness was very similar to that produced by Lorazepam, but presented a clear improvement in therapeutic tolerability [12].

In the present work, the therapeutic effectiveness and tolerability of a G-B standardized extract were compared with an identical pharmaceutical presentation but one containing Alprazolam and administered for 10 weeks in patients with GAD.

## 2. Methods

### 2.1. Treatment Preparation

Plant material that was used for the elaboration of the experimental treatment was obtained from a controlled crop of the species* Galphimia glauca* Cav. (Family Malpighiaceae) located in the state of Morelos, Mexico. A specimen voucher was prepared and deposited at the IMSSM Herbarium for future reference (IMSSM-11061) and was identified by Abigail Aguilar-Contreras, M.Sc., head of the Herbarium. The aerial parts of the plant (10 kg) were dried at room temperature, protected from light and, once dry, were ground in electrical equipment until obtaining particles of <5 mm. The plant material was degreased with hexane and subsequently extracted in 60% ethanol. The product was separated by partition method in ethyl acetate and water, and the organic part was selected for obtaining a rich Galphimine-B fraction by means of a gravitational chromatographic open column. The final yield of the extract was 23.6%. The product was subjected to analysis by means of HPLC in order to know its content of G-B and used for the elaboration of the experimental treatment.

### 2.2. Treatment Standardization

**Figure 1:**
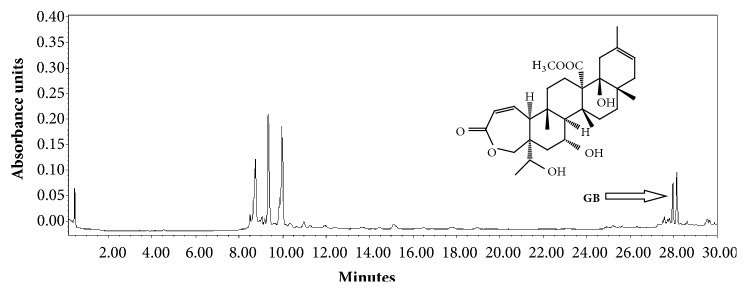
Chromatographic profile of the Galphimine-B (G-B) standardized extract from* Galphimia glauca*. Fingerprint was carried out at 230* n*m and G-B displayed a retention time of 27.97 min. HPLC run conditions are described in methods section.

The dry extract of* Galphimia glauca* was analyzed in a modular HPLC system (Waters) constituted of a 2695 separation model (Alliance; Waters) and a 2996 photodiode detector model (Waters). The equipment was controlled with a data-capture computer-software program (Empower 3; Waters). The chromatographic method was developed in a reverse-phase column (Supelcosil RP-18, 5 *μ*m, 4.6 × 250 mm; Merck). The mobile phase consisted of water (solvent A) and acetonitrile (solvent B). The gradient system was as follows: 0-1 min, 0% B; 2-3 min, 5% B, 4-20 min, 30% B; 21-23 min, 50% B 14-15 min; 24-25min, 80% B; 26-27 100% B; 28-30 min, 0% B. The flow rate was maintained at 0.9 mL/min and the injection volume was 10 *μ*L. The fingerprints were obtained at a 230-*n*m wavelength. For quantitative analysis, previously isolated G-B was used as standard to build a calibration curve. Four ascendant concentrations (0.050, 0.100, 0.200, and 0.400 *μ*g/mL) of this triterpene were injected into the HPLC by triplicated (10 *μ*L). Peak area data obtained at 230* n*m allowed obtaining the calibration curve (Tr=27.97 min, R2=0.99) [13]. This methodology allowed us to know that the* G. glauca* extract contained 53 mg/g of G-B (Figure 1).

### 2.3. Treatments

For the experimental treatment, the product standardized in its G-B content was packed in hard gelatin capsules with the amount necessary to contain 0.374 mg of G-B per dose. For elaboration of the control treatment, Alprazolam was utilized, which was donated by a medical supplier and was accompanied by a Certificate of Quality. Alprazolam was packed in hard gelatin capsules identical to those of the experimental treatment. The capsules were packed in PVC and aluminum blisters with 10 units each. Three blisters, with 10 capsules each, were packed in cardboard boxes. The boxes contained legends with the number of the authorized project, instructions for patients, and a progressive identification number.

### 2.4. Subjects

The study was conducted in a hospital (secondary-level care) belonging to the Mexican Institute of Social Security (IMSS) in the state of Morelos, Mexico. The project was authorized by the National Committee for Scientific Research, by the Ethics Committee, and by the Institutional Biosafety Committee (R-2015-782-112). The study was carried out according to the guidelines of the Helsinki and Tokyo Declarations for humans. Each patient included in the study received detailed information on the clinical procedure and signed a letter of informed consent. A randomized, double-blind, controlled study was performed that included outpatients of both sexes and aged over 35 years, who, by means of the Hamilton Anxiety Scale, showed moderate or severe GAD with a minimal score of 19 points on the previously mentioned scale. Patients with treatment (during the previous month) for the condition, with laboratory data suggestive of liver or kidney damage, pregnant women, or women who were breast feeding, as well as patients with another mental disorder or who did not agree to sign an informed consent letter, were not included in the study. In order to identify another mental disorder, the capture of the patient's medical history was used. It was asked about the presence of mental illnesses, the use of illegal drugs, and the administration of other drugs for the treatment of different diseases.

### 2.5. Study Description

Candidates to participate in the study were evaluated by physicians trained for this purpose. All patients, in order to be included, were required to authorize clinical laboratory tests, and the diagnosis of GAD must have been corroborated, as well as the inclusion criteria.

Through a randomized procedure based on a random number table, patients were assigned to one of the two treatment groups: (1) experimental group, with oral administration of a daily dose in the morning, during 10 weeks, of the G-B standardized extract (0.374 mg/dose), and (2) control group, with administration of Alprazolam (1 mg/dose) under the same conditions and during the same time. It is noteworthy that, in some cases, it was necessary to administer the medication in the evenings (for a two weeks period) due to the needs of the patients.

As mentioned previously, the minimal score (on Hamilton Anxiety Scale) to enter the study was 19 points. At the beginning (baseline) and at the end of the treatment, different scoring ranks were considered as follows: 0-5 = no anxiety or anxiety in remission; 6-18 = mild anxiety; 19-30 = moderate anxiety; 31-42 = severe anxiety; and ≥ 43 = very severe anxiety [13].

In all patients, a follow-up was performed every 2 weeks. In each of the evaluations, patients' adherence to treatment, their general health status, the presence of adverse effects, and the perception of health status were evaluated. The latter was evaluated by means of a Health Scale with the following five response options: 0 = a perception of poor health status; 1 = fair; 2 = good; 3 = very good; and 4 = excellent.

Adverse events were not predefined, only some examples were provided to patients, such as insomnia, headache, dizziness, and diurnal drowsiness. For the evaluation of intensity, a Likert scale with the following response options was used: (1) mild: the adverse effect is present, but it is easily tolerated and does not require treatment; (2) moderate: the adverse effect is enough to interfere with normal activities; (3) severe: the adverse effect incapacitates the subject; (4) serious: it is any adverse effect that results in the death of the patient, endangers life, requires hospitalization, causes disability or persistent or significant disability, and causes a congenital anomaly or cancer, requiring surgical intervention to prevent permanent sequel or develop drug dependence or abuse.

The Health Scale was also employed to evaluate the mental health state in each of the patients included in the two study groups. The following questions were included: (1) after your last physician visit, did your condition make it difficult for you to work inside or outside the home? (options 0 = no; 1 = a little bit; 2 = a fair amount; 3 = fairly; 4 = quite a lot); (2) did you stop engaging in daily activities because of sadness, nervousness, or depression? (options 0 = no; 1 = a little bit; 2 = a fair amount; 3 = fairly; 4 = quite a lot); (3) did you take less care in your daily activities due to sadness, nervousness, or depression? (options 0 = no; 1 = a little bit; 2 = a fair amount; 3 = fairly; and 4 = quite a lot); (4) after your last physician visit, how long did you feel calm and peaceful? (options 1 = always; 2 = nearly always; 3 = often; 4 = some-times; 5 = never); and (5) after your last physician visit, did you feel discouraged and sad? (options 1 = never; 2 = some-times; 3 = often; 4 = nearly always; and 5 = always).

### 2.6. Outcome Variables

Once the 10 weeks of treatment administration was completed, all patients underwent a final evaluation to define the output variables: (1) therapeutic effectiveness, considered when the GAD was completely resolved, or when a condition became less severe; (2) therapeutic failure, considered when the patient did not improve to an anxiety condition of lesser severity or when it was necessary to withdraw the patient from the study due to the adverse effects produced by the administration of the treatment, and (3) therapeutic success was considered when the patient presented therapeutic effectiveness plus the absence of adverse effects that had been the cause of the patient's withdrawal from the study.

One month after completing treatment administration, the patients were invited to participate in a relapse assessment. Relapse was considered when the signs or symptoms of GAD were again present in the patient who had concluded as asymptomatic. When patient concluded treatment administration with some degree of anxiety, the Hamilton Anxiety Scale was used, and it was considered an increase in the intensity when the scale value changed to a higher intensity stage.

### 2.7. Statistical Analysis

Results were analyzed by means of descriptive statistics and expressed as frequencies and percentages. The X^2^ test was used to analyze differences in proportions and Analysis of Variance (ANOVA) for mean differences, as well as the Tukey test for analyzing the differences between groups. Values of p <0.05 were utilized to define significant differences between the treatment groups.

## 3. Results

A total of 167 patients were included in the study and were divided into two study groups: an experimental group that consisted of 84 patients, and a control group in which 83 participants were included. Of all patients, 82.1% (69) of the experimental group and 79.5% (66) of the control group completed 10 weeks of administration. During the development of the study, six (3.5%) patients from the experimental group and six (3.5%) from the control group withdrew from the study because of personal reasons unrelated to the clinical study. Due to the presence of other health problems (broken bone, sprains, low back pain, arthritis, cholecystitis, and tumors), different from the disease-under-study, five (5.9%) patients in the experimental group and one (1.2%) in the control group had to leave the study.

In all patients (167), the presence of adverse effects was analyzed. This variable was present in 67.8% (57) of the patients included in the experimental group and in 95.1% (79) of those of the control group, evidencing a statistically significant difference between groups (p<0.001). Regarding the severity and duration of the adverse effects, in the experimental group (in which patients were treated with the G-B standardized extract), 73.6% of cases were of mild intensity and lasted 1 week or less. In the case of patients in the control group (who received Alprazolam) moderate (52.5%) intensity predominated of the adverse effects and duration was 1 week or more. In both groups, it was necessary to withdraw patients from the study due to the presence of adverse effects that, although not serious, patients reported not being able to carry out their activities and work adequately. In all cases, the adverse effect for which they had to leave the study was daytime sleepiness. For this reason, four (7%) and 10 (12.6%) patients, from the experimental and control group, respectively, had to leave the study. Statistical analysis demonstrated a significant difference (p<0.05) between the two study groups. Other patients presented daytime sleepiness of lesser intensity; thus, it was not necessary for them to leave the study. In this regard, this adverse effect was informed by 4.7% of the patients treated with the G-B standardized extract and in 22.2% of the patients who received Alprazolam.

The mean age of the patients included was 52 years, and women predominated, comprising 88.6%. The evolution time of GAD in patients was of 30 ± 13 months in the control group and 36 ±19 months in the experimental group. Other variables related to personal aspects of the participants are described in Table 1.

Before initiating treatment administration and in terms of the baseline condition, the Hamilton Anxiety Scale was performed in all patients. In the experimental group, the average score was 35.8 ± 8.1 and in the control group, 35.1 ± 8.8, while at the end of the treatment, the score decreased to 3.5 ± 5.5 and 4.6 ± 6.5, in the same order. In all of the patients and on each of their physician visit, a self-evaluation was carried out by means of a Health Scale.  Table 2 depicts the average response of the patients; a progressive and consistent increase in the perception of health improvement can be observed. The perception of an excellent state of health increased significantly, while the perception of a fair and poor state of health declined, nearly disappearing. Statistical analysis of the results, in this case, did not demonstrate significant differences between the study groups and on each of the evaluations. In all cases, both in the experimental group and in the control group, a consistent reduction in the patients' anxiety was observed when the period of treatments administration ended. No statistical difference was found between the groups.

Within the Health Scale, questions that corresponded to mental health status were included. In this part of the survey, as presented in Table 2, a gradual improvement can be observed, which was proportional to the time elapsed between administration of the treatments. This scale exhibits evidence of an improvement in the ability of patients to perform their life activities, both at home and at work. This improvement also coincides with the perception of improvement in other important aspects of the disease and mood, by means of decreasing the patients' perception of nervousness, sadness, and depression. Patients also have a perception of improvement because they feel increasingly calm.

The analysis of results at the end of the study showed that, of the total number of patients included and who finished the 10 weeks of administration, 68.1% (47) from the experimental group reached the status of asymptomatic and 31.8% (22) proceeded to a less severe condition, while in the control group, 57.5% (38) were asymptomatic and 42.4% (28) of the patients had a less severe anxiety condition (p=0.27). Therapeutic success in the experimental group was reached in 92.0% (69) of the patients, while this condition was achieved in 85.7% (66) of the patients in the control group. We observed therapeutic failure in 8.0% and in 14.2% of patients, in the same order (p=0.21).

Regarding the perception of patient satisfaction in response to administration of the treatments, this was 96.2% in the experimental group and 88.6% in the control group (p=0.07).

In order to evaluate relapses, 97.1% (66) of experimental group patients and 95.45% (62) of control group patients visited our physician 1 month later after the finished the administration period. A total of 68.6% (46) of patients in the experimental group reported being asymptomatic, while this condition occurred in 55.5% (35) of patients in the control group. There was no statistically significant difference between the study groups (p=0.39).

## 4. Discussion

In a previous clinical trial, a treatment conducted with the standardized extract obtained from the species Galphimia glauca had shown therapeutic effectiveness in the treatment of patients with GAD [12]. In the latter study, administration of the treatments was carried out for 4 weeks, and Lorazepam (1 mg/dose) was utilized as control treatment. In the present study, a pharmaceutical preparation was elaborated and standardized on G-B content (an anxiolytic compound from* Galphimia glauca*), which was administered daily for 10 weeks and compared, in this case, with Alprazolam. Both Lorazepam and Alprazolam constitute drugs that have been employed for many years in Medicine for the treatment of anxiety. Both are powerful anxiolytics that belong to the group of benzodiazepines; however, these drugs differ in some aspects of their chemical structure and in some characteristics of their pharmacological effect. Alprazolam, in clinical practice, is better indicated in the treatment of GAD, because it has a shorter half-life and, especially, in that the diurnal sleepiness that it produces (adverse effect) occurs in a smaller number of patients, and with less intensity. For this reason, in this study it was particularly important to contrast the effectiveness and tolerability of the experimental treatment with Alprazolam. In the results, it was observed that the G-B standardized extract was effective in consistently reducing the anxiety state of the patients: 68.1% of the patients were completely asymptomatic at the end of the administration of the experimental treatment, and 92.0% of patients were considered with therapeutic success. Although there was no statistical difference between the two treatment groups with respect to this variable, a lower percentage (85.7%) of patients treated with Alprazolam achieved therapeutic success.

Also in this study, different procedures were employed to follow the evolution of patients, and a self-evaluation was utilized in which the patient described, at each of his physician visits, the perception of his/her general health status, and specifically, their mental health status. With the obtained results, it was possible to appreciate that the perception of satisfaction of the patients in response to the administration of the treatments was higher in the patients included in the experimental treatment (96.2%) than in those in the control group (88.6%). The self-evaluation also revealed that the administered treatments managed to progressively improve the activities of patients in daily life, as well as provide palpable improvements regarding their tranquility and their perception of anxiety and depression. In this respect, effective treatment of anxiety disorder allows the patient to better resolve emotional conflicts and achieve a better performance in their family, social, and work lives. In this study, it was possible to identify that the most important difference between the treatments administered was found in the adverse effects, particularly in daytime sleepiness, which was present in a greater number of patients in the group treated with Alprazolam.

It is evident that the mechanism of action of G-B differs ostensibly from that presented by Alprazolam and other benzodiazepines. Previous studies have demonstrated that G-B does not interact with the GABAergic system and that it shows selectivity for specific areas of the CNS, such as the VTA [10] and the dorsal hippocampus [11]. The mechanism of action of G-B on the dopaminergic system, widely related in the genesis of anxiety and in its specific effects in neuronal systems and regions of the CNS, could be the reason for the differences found in this study. G-B was used as a marker in order to standardize the extract that was used in the preparation of the capsules, however it is necessary to highlight that other Galphimines (G-A and G-E) are also present in the extract and may be contributing to the observed efficacy. Other unidentified compounds are present in the standardized extract, but it is important to note that the amount of extract in each capsule was of 7.056 mg. It is important to take into consideration that in this study (avoiding living patients without medical treatment) a placebo group was not included; this situation does not allow a comparative analysis to identify a possible placebo effect. In addition to the above, it is necessary to point out that in the present study only Alprazolam was used in the control group and that some clinical practice guidelines, in some countries, could recommend the use of more than one drug or other therapeutic procedures.

## 5. Conclusion

It is possible to conclude that a G-B standardized extract (0.374 mg/dose), administered daily for 10 weeks, shows therapeutic effectiveness, tolerability, and safety in patients with GAD. The observed clinical effectiveness did not demonstrate a significant difference between treatment groups; however, the experimental group exhibited fewer cases of daytime sleepiness than the control group.

## Figures and Tables

**Table 1:** Personal characteristics of patients included in the study, organized by treatment group administered. The experimental group was treated with the Galphimine-B (G-B) standardized extract and the control group was administered with Alprazolam.

Variable	Experimental group	Control group	*P*
	*n* = 84	*n* = 83	
Age (years)	52.3±9.3	52±8.8	0.66
	F(%)	F(%)	
**Gender**			
Masculine	8(9.5)	11(13.2)	0.44
Feminine	76(90.4)	72(86.5)	
**Civil status**			
Single	7(8.3)	6(7.2)	
Married	57(67.8)	54(65)	0.71
Common-law marriage	9(10.7)	12(14.4)	
Divorced	7(8.3)	5(6)	
Widowed	4(4.7)	4(4.8)	
Other	0(0)	2(2.4)	
**Scholarship**			
Basic	48(57.1)	43(49.3)	0.54
Upper middle	24(28.5)	23(27.7)	
University	12(14.2)	19(23.1)	
**BMI**			
Underweight	2(2.3)	3(3.6)	0.85
Normal weight	12(14.2)	15(18)	
Overweight	33(39.2)	32(38.5)	
Obesity	37(44)	33(79.7)	
**Working**			
No	46(55.4)	50(60.2)	0.53
Yes	38(45.2)	33(39.7)	

F=frequency; %=percentage; BMI=body mass index.

**Table 2:** Effect produced by oral administration for 10 weeks of a Galphimine-B (G-B) standardized extract (experimental group) or Alprazolam (control group) on perception of mental health status in patients diagnosed with Generalized Anxiety Disorder (GAD) included in the study. The data were obtained through a self-evaluation by means of a Health Scale.

	**Experimental group/Control group**				
Variable	2 weeks	4 weeks	6 weeks	8 weeks	10 weeks
	*n* 76 / 61	*n* 62/58	*n *69/71	*n* 63/62	69/64
	F / (%)	F / (%)	F / (%)	F / (%)	F / (%)
**Perception of the patient's health**					

Excellent	4(5.3) / 1(1.6)	4(6.5) / 4(6.8)	8(11.5) / 14(19.7)	19(30.1) / 16(25.8)	30(43.4) / 23(35.9)

Very good	9(11.8) **/ **13(21.3)	13(20.9) / 11(18.9)	29(42) / 18(25.3)	21(33.3) / 23(37)	21(30.4) / 23(35.9)

Good	35(46) **/ **23(37.7)	34(54.8) / 32(55.1)	24(34.7) / 25(35.2)	16(25.3) / 18(29)	11(15.9) / 14(21.8)

Fair	26(34.3) **/ **23(37.7)	11(17.7) / 11(18.9)	8(11.5) / 13(18.3)	5(7.9) / 4(6.4)	7(10.1) / 4(6.5)

Poor	2(2.6) **/ **1(1.6)	0(0) / 0(0)	0(0) / 1(1.4)	2(3.1) / 1(1.6)	0(0) / 0(0)

**Person X** ^**2**^ **/*p***	**3.93 / 0.41**	**0.29 / 0.96**	**6.99 / 0.22**	**0.90 / 0.92**	**2.00 / 0.57**

**After your last physician visit, did your condition make it difficult for you to work inside or outside the home?**					

No	53(69.8) / 26(42.6)	40(64.5) / 33(56.8)	52(75.4) / 39(54.9)	48(76.2) / 43(69.3)	55(79.7) / 44(68.7)

A little bit	21(27.7) / 22(36)	17(27.4) / 18(31)	16(23.2) / 27(38)	15(23.8) / 16(25.8)	14(20.2) / 16(25)

A fair amount	2(2.6) / 6(9.8)	5(8) / 6(10.3)	1(1.5) / 5(7)	0(0) / 2(3.2)	(0) / 3(4.6)

Fairly	0(0) / 3(4.9)	0(0) / 1(1.7)	0(0) / (0)	0(0) / 0(0)	(0) / 1(1.5)

Quite a lot	0(0) / 4(6.5)	0(0) / (0)	0(0) / (0)	0(0) / 1(1.6)	(0) / (0)

**Person X** ^**2**^ **/*p***	**16.81 / 0.002**	**1.65 / 0.64**	**7.31 / 0.02**	**3.29 / 0.34**	**5.17 / 0.15**

**Did you stop engaging in daily activities because of sadness, nervousness or depression?**					

No	50(65.7) / 28(45.9)	44(70.9) / 32(55.1)	51(73.9) / 40(56.3)	47(74.6) / 42(67.7)	54(78.6) / 47(73.4)

A little bit	19(25) / 19(31.1)	15(24.1) / 16(27.5	17(24.6) / 28(39.4)	15(23.8) / 18(29)	14(20.2) / 16(25)

A fair amount	4(5.2) / 7(11.4)	3(4.8) / 9(15.5)	1(1.4) / 3(4.2)	1(1.5) / 1(1.6)	1(1.4) / 0(0)

Fairly	2(2.6) / 4(6.5)	0(0) / 1(1.7)	0(0) / 0(0)	0(0 ) / 1(1.6)	0(0) / 1(1.5)

Quite a lot	1(1.3) / 3(4.9)	0(0) / 0(0)	0(0) / 0(0)	0(0) / (0)	0(0) / (0)

**Person X** ^**2**^ **/*p***	**7.13 / 0.12**	**5.8 / 0.12**	**4.99 / 0.08**	**1.54 / 0.67**	**2.4 / 0.48**

**Did you take less care in your daily activities due to sadness, nervousness, or depression?**					

No	52(68.4) / 24(39.3)	43(69.3) / 34(58.6)	51(73.9) / 42(69.3)	49(77.7) / 43(69.3)	53(76.8) / 45(70.3)

A little bit	17(22.4) / 23(37.7)	16(25.8) / 16(27.5)	17(20.6) / 25(27.5)	13(20.6) / 17(27.5)	15(21.7) / 18(28.1)

A fair amount	2(2.6) / 8(13.1)	3(4.8) / 7(12)	1(1.5) / 4(5.6)	1(1.5) / 1(1.7)	1(1.4) / 0(0)

Fairly	3(3.9) / 2(3.2)	0(0) / 1(1.8)	0(0) / 0(0)	0(0) / 0(0)	0(0) / 1(1.5)

Quite a lot	0(0) / 1(1.6)	0(0) / 0(0)	0(0) / 0(0)	0(0) / 1(1.7)	0(0) / 0(0)

**Person X** ^**2**^ **/*p***	**14.7 / 0.01**	**3.52 / 0.31**	**4.16 / 0.12**	**1.91 / 0.59**	**2.74 / 0.43**

**After your last physician visit, how long did you feel calm and peaceful?**					

Always	17(22.3) / 25(40.9)	13(20.9) / 15(25.8)	29(42) / 21(29.6)	29(46.7) / 23(37)	46(66.7) / 39(60.9)

Nearly always	29(38.3) / 21(34.4)	33(53.2) / 25(43.2)	30(43.4) / 32(45)	22(34.9) / 31(50)	14(20.2) / 21(32.8)

Often	8(10.5) / 5(8.3)	4(6.5) / 5(8.6)	5(7.3) / 9(12.6)	5(7.9) / 3(4.8)	3(4.4) / 0(0)

Some-times	22(28.9) / 10(16.3)	13(20.9) / 13(22.3)	5(7.3) / 9(12.6)	7(11.1) / 5(8.2)	6(8.7) / 4(6.2)

Never	0(0) / 0(0)	0(0) / 0(0)	0(0) / (0)	0(0) / 0(0)	0(0) / (0)

**Person X** ^**2**^ **/*p***	**6.83 / 0.14**	**2.22 / 0.69**	**5.38 / 0.25**	**3.04 / 0.38**	**5.19 / 0.15**

**After your last physician visit, did you feel discouraged and sad?**					

Never	35(46) / 29(47.5)	28(45.2) / 23(39.6	44(63.7) / 27(38)	44(69.8) / 33(53.3)	50(72.4) / 44(68.7)

Some-times	37(48.6) / 31(50.8)	33(53.1) / 32(55.1)	25(36.1) / 39(54.8)	18(28.5) / 27(43.4)	19(27.4) / 8(12.5)

Often	3(3.9) / 0(0)	1(1.6) / 1(1.7)	0(0) / 4(5.6)	1(1.5) / 0(0)	0(0) / 1(1.5)

Nearly always	1(1.3) / 0(0)	0(0) / 2(3.4)	0(0) / 1(1.4)	0(0) / 2(3.2)	0(0) / 0(0)

Always	0(0) / 1(1.6)	0(0) / 0(0)	0(0) / 0(0)	0(0) / 0(0)	0(0) / 0(0)

**Person X** ^**2**^ **/*p***	**7.91 / 0.16**	**2.56 / 0.63**	**13.76 / 0.008**	**6.24 / 0.18**	**3.64 / 0.30**

F = frequency; % = percentage.

## Data Availability

Data used to support the findings of this study are available from the corresponding author upon request. In order to maintain confidentiality with the patients included in the study, some data would not be available.
